# Targeted sequencing and integrative analysis to prioritize candidate genes in neurodevelopmental disorders

**DOI:** 10.1007/s12035-021-02377-y

**Published:** 2021-04-15

**Authors:** Yi Zhang, Tao Wang, Yan Wang, Kun Xia, Jinchen Li, Zhongsheng Sun

**Affiliations:** 1 National Clinical Research Centre for Geriatric Disorders, Department of Geriatrics, Xiangya Hospital, Central South University, Changsha, 410083 Hunan China; 2 Center for Medical Genetics and Hunan Key Laboratory of Medical Genetics, School of Life Sciences, Central South University, Changsha, 410083 Hunan China; 3 Beijing Institutes of Life Science, Chinese Academy of Sciences, Beijing, 100101 China; 4 DIAGenes Precision Medicine, Beijing, 102600 China; 5 CAS Center for Excellence in Brain Science and Intelligences Technology (CEBSIT), Shanghai, 200031 China; 6 School of Basic Medical Science, Central South University, Changsha, 410078 Hunan China; 7 Department of Neurology, Xiangya Hospital, Central South University, Changsha, 410008 Hunan China; 8 Institute of Genomic Medicine, Wenzhou Medical University, Wenzhou, 325035 China; 9 CAS Center for Excellence in Biotic Interactions, University of Chinese Academy of Sciences, Beijing, 100049 China; 10 State Key Laboratory of Integrated Management of Pest Insects and Rodents, Chinese Academy of Sciences, Beijing, 100101 China

**Keywords:** Neurodevelopmental disorders, Targeted sequencing, De novo variants, Candidate genes

## Abstract

Neurodevelopmental disorders (NDDs) are a group of diseases characterized by high heterogeneity and frequently co-occurring symptoms. The mutational spectrum in patients with NDDs is largely incomplete. Here, we sequenced 547 genes from 1102 patients with NDDs and validated 1271 potential functional variants, including 108 de novo variants (DNVs) in 78 autosomal genes and seven inherited hemizygous variants in six X chromosomal genes. Notably, 36 of these 78 genes are the first to be reported in Chinese patients with NDDs. By integrating our genetic data with public data, we prioritized 212 NDD candidate genes with FDR < 0.1, including 17 novel genes. The novel candidate genes interacted or were co-expressed with known candidate genes, forming a functional network involved in known pathways. We highlighted *MSL2*, which carried two de novo protein-truncating variants (p.L192Vfs*3 and p.S486Ifs*11) and was frequently connected with known candidate genes. This study provides the mutational spectrum of NDDs in China and prioritizes 212 NDD candidate genes for further functional validation and genetic counseling.

**Supplementary Information:**

The online version contains supplementary material available at 10.1007/s12035-021-02377-y.

## Introduction

Neurodevelopmental disorders (NDDs) are complex and heterogeneous disorders characterized by impaired motor function, learning, and verbal and non-verbal communication resulting from dysfunction during brain development [1–4]. NDDs span a very wide range of neurological and psychiatric disorders [1, 5] and affect more than 3% of children worldwide [6]. Although each diagnosis is distinct in clinical settings, in general, NDDs are characterized by high clinical and genetic heterogeneity. Previous studies have estimated the heritability of NDDs, with the highest being up to ~ 80% [7–10]. Multiple phenotype-genotype correlation studies have revealed that patients carrying deleterious variants in the same risk gene have manifested broad clinical phenotypes, including impaired social interaction, developmental delay, regression, and repetitive behavior [11–13]. The high level of heterogeneity and similar pathogenetic mechanisms present challenges for clinical diagnosis and treatment.

Recent studies have reported that targeted sequencing is a powerful and cost-effective tool for discovering new genetic risk genes in many diseases, particularly diseases with high genetic heterogeneity [4, 14, 15]. Both rare inherited and de novo variants (DNVs) have been demonstrated to contribute to NDDs [16–18]. Stessman et al. sequenced 208 candidate genes in patients with NDDs and identified 91 risk genes with locus-specific significance for disruptive variants in 5.7% of patients [4]. Wang et al. performed targeted sequencing of 189 autism spectrum disorder (ASD) candidate risk genes in Chinese patients and found that genes identified in European ASD cohorts were highly relevant in Chinese cohorts [19]. Guo et al. expanded the above study to a larger cohort of patients and provided support for a multifactorial model of ASD risk [20]. Some studies have shown that there are multiple risk genes that are shared between neurodevelopmental disorders, and integrating datasets at multiple levels is beneficial for the etiology of NDDs [21, 22]. Takata et al. combined published DNV data and found that integrative analyses was conducive to identifying significant genes and extending ASD-related molecular and brain networks [23]. Gonzalez-Mantilla et al. used multilevel data-integration approach and identified novel candidate genes for developmental brain disorders [24]. We have previously demonstrated that DNVs, gene set enrichment analysis, and protein-protein interaction (PPI)/co-expression analysis can provide new insights into the genetic mechanisms underpinning NDDs [25–28]. However, only a few known pathogenetic genes have been described to explain the genetic causes of NDDs, and the etiology behind NDDs remains unclear.

Here, we used targeted sequencing to examine 547 genes from 1102 Chinese patients with NDDs. We identified potential functional variants and explored the patterns of DNVs in Chinese patients with NDDs. We then integrated our dataset with public datasets of neurodevelopmental disorders from the Gene4Denovo [29] database to prioritize NDD candidate genes and discover novel candidate genes. Finally, we investigated whether novel candidate genes were functionally associated with known candidate genes using multilevel bioinformatics analysis.

## Materials and Methods

### Panel Gene Design and Targeted Sequencing

In this study, potential NDD risk genes are collected based on the following criteria: (1) genes recorded in the Online Mendelian Inheritance in Man (OMIM) (https://omim.org/) are associated with NDDs, mainly including autism, cerebral palsy, mental retardation, and epilepsy; (2) candidate genes in the NPdenovo database [28]; (3) strong risk genes from the SFARI Gene database (https://gene.sfari.org/) [30]; and (4) genes prioritized in our previous NDD-related studies [25, 26, 31, 32]. After removing duplicated genes, 547 target genes were selected (Table S1).

A total of 935 unrelated trios (probands and their unaffected parents) and 167 probands without parents were recruited from China. Genomic DNA (1 μg) extracted from whole blood was sheared and assembled into a DNA library prior to targeted sequencing. The Illumina X10 sequencing system (Illumina, San Diego, CA, USA) was used to generate paired-end raw data. This panel resulted in an average depth of 181.46× in target regions, and 98.82% of target bases were covered with depth ≥ 10× on average. This study was approved by the Institutional Review Board of the State Key Laboratory of Medical Genetics, School of Life Sciences, Central South University, Changsha, Hunan, China. All subjects who participated in this study provided informed consent prior to sample collection.

### Variation Detection and Annotation

Quality control of the sequencing data was performed using Cutadapt [33] and FastQC (https://www.bioinformatics.babraham.ac.uk/projects/fastqc/) to remove adapter and unqualified sequences, respectively. BWA-MEM [34] was employed to align the clean reads to the human reference genome (hg19). Samtools [35] utilities were used to mark duplicate reads and generate position-sorted files, while the Genome Analysis Toolkit HaplotypeCaller was used to call variants. Comprehensive annotation of all variants was performed using ANNOVAR [36], including functional implications (gene region, functional effect, mRNA GeneBank accession number, amino acid change, cytoband, etc.), functional predictions for missense variants, and allele frequencies of gnomAD, ExAC, and in-house data (1113 WES samples and 2469 WGS samples). Deleterious missense variants (Dmis) were predicted by ReVe [37] and missense variants with ReVe ≤ 0.7 were excluded. Only protein-truncating variants (PTVs, including stop-gain, stop-loss, frameshift, and splicing) and Dmis with minor-allele frequencies ≤ 0.1% were defined as potential functional variants and selected for further analysis. Sanger sequencing was used to validate all potential functional variants in our study.

### Prioritization of Candidate Genes

Using de novo PTVs, together with Dmis and background DNV rates, we employed the TADA classification tool [38] to prioritize candidate genes, with gene exhibiting a false discovery rate (FDR) < 0.1 defined as candidates. First, DNVs from 935 trios, inherited variants from 1102 probands, and genetic variants from 3582 in-house Chinese controls (Table S6) were applied to TADA model, and we prioritized 17 candidate genes with FDR values < 0.1. To increase the statistical power, we integrated DNVs from 16,807 probands with different types of NDDs and included 3391 controls from the Gene4Denovo database (version 1.0) [39] and 208 candidate genes with FDR values < 0.1 for further analysis. All samples integrated in our study have been carefully deduplicated.

To discover novel candidate genes, we collected known candidate genes from previous studies and related databases, and excluded them from the list of candidate genes prioritized by TADA. Known candidate genes were defined as follows: (1) genes defined as risk genes in any ten recent publications involving large-scale exome, whole-genome, or targeted sequencing (De Rubeis et al. [17], Sanders et al. [40], Lelieveld et al. [41], McRae et al. [42], Stessman et al. [43], Yuen et al. [44], Nguyen et al. [22], Takata et al. [23], Coe et al. [21], and Satterstrom et al. [45]); (2) genes collected from OMIM that were associated with neurodevelopmental disorders, including intellectual developmental disorder, autism, mental retardation, epileptic encephalopathy, and schizophrenia; (3) genes belonging to the category of syndromic gene or score category of 1 or 2 in SFARI Gene [30]. Genes that met any of the above conditions were classified as known candidate genes. In contrast, genes that failed to meet any of the above conditions above and had no obvious genetic evidence in association with NDDs in PubMed were classified as novel candidate genes.

### Permutation Test

Spatial and temporal expression data of the human brain were downloaded from BrainSpan (http://www.brainspan.org/). For ethical reasons, we removed samples during fetal stages and selected postnatal cortical samples for further study. We calculated the Pearson correlation coefficients between any two genes based on their expression levels. Gene pairs with |*R*| > 0.7 were regarded as being co-expressed in the human brain. The permutation test was performed to compare the novel and the known candidate gene sets, in order to evaluate their functional connections. In brief, we compared the number of co-expressed genes within the novel and the known candidate gene sets and their connections with a random gene set of 1,000,000 random iterations.

### Functional Network Analysis

We downloaded PPI data from IntAct Molecular Interaction database (https://www.ebi.ac.uk/intact/) for analysis and considered gene pairs with an intact-miscore ≥ 0.45 to be interacting genes. We then constructed a network based on gene pairs selected from our PPI and co-expression analyses. Novel candidate genes were defined as “seed genes” that were directly connected and used to form an interconnected functional network. Known NDD candidate genes directly connected to at least two novel candidate genes were added to the above network. To further investigate the functional pathways of novel candidate genes, we performed GO enrichment analysis using MetaScape (https://metascape.org). Similar pathways were merged into a single cluster. Network figures were drawn using Cytoscape v.3.7.2.

## Results

### DNVs in Our Chinese Cohort

**Fig. 1:**
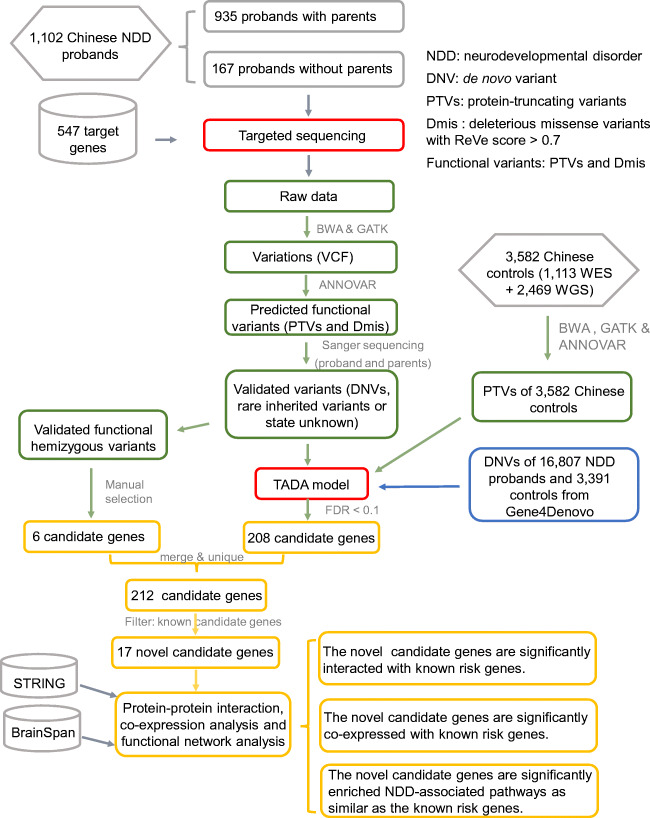
Study workflow. This study consisted of four parts: (1) sample collection; (2) identification and validation of variants; (3) prioritization of candidate genes; (4) functional network analysis of novel and known candidate genes. PTVs, protein-truncating variants; Dmis, deleterious missense variants

In this study, we sequenced 547 target genes in 1102 Chinese patients with NDDs and identified a set of predicted potential functional variants, including PTVs and Dmis (Fig. 1 and Table S1). Using Sanger sequencing, we successfully validated 1271 potential functional variants, including 108 de novo variants (54 de novo PTVs and 54 de novo Dmis), 975 inherited variants (156 inherited Dmis and 819 inherited PTVs), and 188 undetermined variants (36 PTVs and 152 Dmis) (Fig. S1 and Table S2). We found that 108 DNVs in 78 genes appeared in approximately 10.91% (102/935) of patients in our Chinese cohort. Among these 78 genes, 36 genes with DNVs are the first to be reported in Chinese patients. Furthermore, we revealed that 21 genes carried multiple DNVs, including 28 de novo PTVs and 23 de novo Dmis (Table 1). Both *SCN2A* and *MECP2* were the most frequently mutated genes, each carrying one de novo PTV and three de novo Dmis (Table 1). Five genes (*MED13L*, *GRIN2B*, *KCNQ2*, *CTNNB1*, and *TCF20*) carried three DNVs in our Chinese cohort, while another 14 genes (*ASH1L*, *SATB2*, *NRXN1*, *BCL11A*, *ADNP*, *SHANK3*, *MSL2*, *SYNE1*, *SYNGAP1*, *BRAF*, *GATAD2B*, *LLGL1*, *SLC2A1*, and *KDM5C*) carried two DNVs (Table 1). We manually collected 805 known candidate genes that have either been reported in studies on large NDD cohorts or have strong evidence associating them with NDDs in the OMIM or SFARI Gene databases [17, 21–23, 40–45] (Table S3). We found that of 21 genes with multiple DNVs that we identified, 20 are classified as known candidate genes (Table S4). For example, *TCF20*, which carries three *de novo* PTVs (p.S1803Vfs*6, p.C1795Wfs*13, and p.R1907X), was first reported in a Chinese cohort. Interestingly, we identified a potential novel NDD risk gene (*MSL2*) that carries two *de novo* PTVs (p.S560Ifs*11 and p.L266Vfs*3). This study is the first to report an association between *MSL2* and NDDs.

**Table 1:** Genes with multiple DNVs in our probands

Gene symbol	DNVs (*n* = 935)	Inherited or state unknown (*n* = 1102)	DNVs from Gene4Denovo (*n* = 16,807)	RVIS (percentile)	pLI (percentile)	Gene function
*SCN2A*	1 PTV, 3 Dmis	1 PTV, 3 Dmis	18 PTVs, 32 Dmis	− 2.51 (1.05%)	1.00 (0.72%)	Nervous system development
*MECP2*	1 PTV, 3 Dmis	1 Dmis	9 PTVs, 10 Dmis	− 0.32 (30.25%)	0.70 (24.62%)	Chromatin binding
*MED13L*	3 PTVs	2 Dmis	18 PTVs, 11 Dmis	− 1.61 (3.34%)	1.00 (0.57%)	Brain development
*GRIN2B*	1 PTV, 2 Dmis	2 Dmis	4 PTVs, 15 Dmis	− 2.48 (1.10%)	1.00 (2.19%)	Nervous system development
*KCNQ2*	1 PTV, 2 Dmis	1 PTV, 3 Dmis	2 PTVs, 25 Dmis	− 1.25 (5.97%)	1.00 (5.39%)	Regulation of neuronal excitability
*CTNNB1*	3 PTVs	1 Dmis	19 PTVs	− 1.07 (8.10%)	1.00 (4.20%)	Wnt signaling
*TCF20*	3 PTVs	3 Dmis	11 PTVs	− 3.82 (0.34%)	1.00 (2.84%)	Wnt signaling
*ASH1L*	2 PTVs	3 Dmis	7 PTVs	− 3.89 (0.31%)	1.00 (0.16%)	Chromatin organization
*SATB2*	2 PTVs	-	11 PTVs, 5 Dmis	− 1.31 (5.37%)	1.00 (4.97%)	Chromatin binding
*NRXN1*	2 Dmis	3 PTVs, 11 Dmis	2 PTVs, 5 Dmis	− 1.88 (2.30%)	1.00 (3.22%)	Nervous system development
*BCL11A*	2 PTVs	1 Dmis	6 PTVs, 3 Dmis	− 1.67 (3.07%)	0.83 (20.63%)	Brain development
*ADNP*	2 PTVs	5 Dmis	26 PTVs, 1 Dmis	− 1.54 (3.72%)	1.00 (5.98%)	Chromatin binding
*SHANK3*	2 PTVs	2 PTVs	9 PTVs	-	1.00 (4.13%)	Nervous system development
*MSL2*	2 PTVs	1 Dmis	1 PTV	− 0.72 (15.01%)	0.89 (18.19%)	Chromatin organization
*SYNE1*	2 Dmis	4 PTVs, 16 Dmis	3 Dmis	− 1.10 (7.70%)	3.75E-27 (99.45%)	Nucleotide binding
*SYNGAP1*	1 PTV, 1 Dmis	1 Dmis	31 PTVs, 1 Dmis	− 2.30 (1.36%)	1.00 (1.79%)	Postsynaptic signaling
*BRAF*	2 Dmis	1 Dmis	10 Dmis	− 0.97 (9.63%)	1.00 (2.83%)	Calcium ion binding
*GATAD2B*	2 PTVs	2 Dmis	11 PTVs	− 0.65 (16.92%)	1.00 (6.34%)	Chromatin remodeling
*LLGL1*	2 Dmis	2 Dmis, 2 PTV	1 Dmis	− 1.70 (2.94%)	0.98 (12.10%)	Axon development
*SLC2A1*	2 Dmis	1 Dmis	4 PTVs, 1 Dmis	− 0.92 (10.49%)	0.94 (15.56%)	Brain development
*KDM5C*	2 Dmis	-	3 PTVs, 2 Dmis	− 2.71 (0.88%)	1.00 (7.02%)	Chromatin organization

*PTVs*, protein-truncating variants, including frameshift, splicing, stop-gain, and stop-loss; *Dmis*, deleterious missense variants with ReVe score > 0.7

We then employed probability of loss-of-function intolerance (pLI) [46] analysis from ExAC and residual variation intolerance scores (RVIS) [47] to evaluate the functional impact of genes with multiple DNVs. According to the scores of pLI and RVIS, we ranked them and used percentiles as an indicator of gene intolerance. Genes with low-percentile RVIS or pLI were more likely to be intolerant to genetic variants. As expected, it was shown that genes with multiple DNVs both exhibited significantly lower percentile pLI (*p* = 4.10 × 10^−10^, two-tailed Wilcoxon rank-sum test) and RVIS (*p* = 1.55 × 10^−10^, two-tailed Wilcoxon rank-sum test) than background genes (Fig. S2a). In particular, 20 of the 21 multiple DNV genes ranked in the top 50% of pLI and RVIS (Table 1), suggesting that they are less tolerant of damaging variants.

### Inherited X-Linked Hemizygous Variants

We identified seven inherited X-linked hemizygous variants in six genes (Table S5). Among these seven variants, three variants, including two Dmis (p.R270C on *PTCHD1* and p.R197H on *SLC16A2*) and one splicing variant (c.1030-1G>C on *SLC9A7*), were recorded in the dbSNP or gnomAD database. The remaining four variants, including three Dmis (p.Q153R on *ATP6AP2*, p.R287C on *ARHGEF9*, and p.C517Y on *PLXNA3*) and one frameshift (p.N136Kfs*30 on *SLC16A2*) are reported here for the first time. Compared with the 805 known candidate genes, we note that five of the above genes are classified as known candidate genes and three (*PTCHD1*, *ATP6AP2*, and *SLC9A7*) are reported for the first time in Chinese patient with NDDs. In addition, we identified a novel candidate gene (*PLXNA3*) carrying a novel hemizygous missense variant (p.C517Y). A previous study has described an ASD patient from Maghreb carrying a rare inherited missense variant (p.D863E) in *PLXNA3* [48], suggesting that it may contribute to autism.

### Prioritization of Candidate Genes

Previous studies have demonstrated that both DNVs and rare inherited variants (RIVs) contribute significantly to NDDs [49–54]. Therefore, we integrated data on DNVs and RIVs and employed TADA [38] to prioritize candidate genes in NDDs (Table S6). Based on DNVs and RIVs identified in our study, we prioritized 17 candidate genes (*SCN2A*, *KCNQ2*, *SATB2*, *SHANK3*, *GATAD2B*, *NRXN1*, *MED13L*, *SYNGAP1*, *BRAF*, *MECP2*, *GRIN2B*, *TCF20*, *CTNNB1*, *ASH1L*, *ADNP*, *BCL11A*, and *LLGL1*) with FDR values < 0.1 (Table S7). To increase the power of candidate gene detection [17, 22, 43, 55–57], we integrated data from public datasets of NDD cohorts (*N* = 16,807) with that of controls (*N* = 3391) from the Gene4Denovo [39] database (Table S8), and prioritized 208 candidate genes with FDR values < 0.1, in which 193 candidate genes reached an FDR < 0.05 (Table S9). Together with the six genes characterized by inherited X-linked hemizygous variants, in total, we prioritized 212 NDD candidate genes, with 17 genes defined as novel candidate genes (including 13 genes with FDR values < 0.05, three genes with 0.05 ≤ FDR < 0.1 (Table 2), and one gene with an inherited X-linked hemizygous variant). We observed that the 212 candidate genes prioritized in our study exhibited significantly lower percentile pLI scores (*p* < 2.20 × 10^−16^, two-tailed Wilcoxon rank-sum test) and RVIS (*p* < 2.20 × 10^−16^, two-tailed Wilcoxon rank-sum test; Fig. S2b), consistent with the result regarding genes with multiple DNVs. A similar result was observed among the 17 novel candidate genes (*p* = 0.0394 and *p* = 1.74 × 10^−4^; two-tailed Wilcoxon rank-sum test; Fig. S2c), suggesting that these novel NDD candidate genes were likely to be intolerant of functional variants.

**Table 2:** Novel candidate genes prioritized by TADA analysis

Gene symbol	DNVs (*n* = 935)	Inherited or state unknown (*n* = 1102)	DNVs from Gene4Denovo (*n* = 16,807)	FDR	RVIS (percentile)	pLI (percentile)	Gene summary
*SMAD6*	-	1 Dmis	3 PTVs, 1 Dmis	4.83E−04	-	6.97E−06 (78.89%)	Developmental and cellular process
*SPG7*	-	3 PTVs, 3 Dmis	5 Dmis	8.89E−04	− 0.91 (10.60%)	1.38E−18 (98.28%)	Anterograde axonal transport
*MSL2*	2 PTVs	1 Dmis	1 PTV	9.62E−04	− 0.72 (15.01%)	0.90 (18.20%)	Chromatin organization
*CYP27C1*	-	2 PTVs, 3 Dmis	1 PTV, 3 Dmis	1.39E−03	− 0.89 (11.06%)	1.85E−04 (70.01%)	Retinal metabolic process; retinol metabolic process
*ITSN1*	-	2 Dmis	3 PTVs, 2 Dmis	1.89E−03	− 2.89 (0.76%)	1.00 (0.87%)	Regulation of modification of postsynaptic actin cytoskeleton
*PSD3*	-	1 PTV, 2 Dmis	3 PTVs, 1 Dmis	2.11E−03	0.09 (56.40%)	0.85 (19.80%)	Nervous system development
*POLR3A*	1 Dmis	5 Dmis	2 PTVs, 1 Dmis	2.99E−03	− 2.26 (1.40%)	7.39E−14 (96.18%)	Nervous system development
*UBR3*	-	-	3 PTVs	5.41E−03	-	1.00 (5.02%)	Chromatin organization; nervous system development
*GALNT18*	-	2 Dmis	2 PTVs, 1 Dmis	7.34E−03	− 1.07 (8.11%)	4.77E−02 (47.21%)	Metabolism of proteins
*DCX*	-	-	2 PTVs, 1 Dmis	7.43E−03	− 0.67 (16.20%)	0.86 (19.34%)	Axoneme assembly
*LRRC4*	-	-	1 PTV, 2 Dmis	2.56E−02	− 0.84 (11.90%)	0.88 (18.68%)	Nervous system development
*SPAG9*	1 PTV	3 Dmis	1 PTV	3.15E−02	− 1.20 (6.59%)	1.00 (1.67%)	Spinocerebellar ataxia 35
*ST3GAL6*	-	-	2 PTVs	3.23E−02	0.07 (55.10%)	1.06E−03 (64.45%)	Pre-NOTCH Expression and processing
*YTHDC1*	-	-	2 PTVs	8.48E−02	− 0.61 (18.16%)	1.00 (1.0864%)	Chromatin regulation/acetylation
*RRAGC*	-	1 Dmis	2 Dmis	9.00E−02	− 0.25 (34.05%)	0.602 (27.41%)	Regulation of TORC1 signaling
*DNAH17*	1 Dmis	3 PTVs, 16 Dmis	1 PTV, 3 Dmis	9.58E−02	-	-	Developmental process

*PTVs*, protein-truncating variants, including frameshift, splicing, stop-gain, and stop-loss; *Dmis*: deleterious missense variants with ReVe score > 0.7

In addition, we found that DNVs and inherited X-linked hemizygous variants in 212 candidate genes were identified in ~ 10.59% (99/935) and ~ 0.94% (7/745) of patients in our study, respectively (Table 3). DNVs and inherited X-linked hemizygous variants in known candidate genes accounted for ~ 10.05% (94/935) and ~ 0.81% (6/745) of patients, whereas in novel candidate genes, these variants accounted for ~ 0.53% (5/935) and ~ 0.13% (1/745) of patients, respectively. Inherited or state unknown variants in all candidate genes were detected in ~ 35.75% (394/1102) of all patients (Table 3).

**Table 3:** The contribution of prioritized candidate genes to our Chinese probands

Class	Known candidate genes (*n* = 195)	Novel candidate genes (*n* = 17)	
FDR_TADA_combined_ < 0.05 (*n* = 193)	180	13 genes: *MSL2*, *SPAG9*, *POLR3A*, *CYP27C1*, *DCX*, *GALNT18*, *ITSN1*, *LRRC4*, *PSD3*, *SMAD6*, *SPG7*, *ST3GAL6*, *UBR3*	
0.05 ≤ FDR_TADA_combined_ < 0.1 (*n* = 15)	12	3 genes: *DNAH17*, *RRAGC*, *YTHDC1*	
Genes with X-linked hemizygous (*n* = 6)	5	1 gene: *PLXNA3*	
Chinese probands with DNVs (*n* = 935)	94 (10.05%)	5 (0.53%)	Subtotal: 99 (10.59%)
Chinese probands with X-linked hemizygous variants (*n* = 745)	6 (0.81%)	1 (0.13%)	Subtotal: 7 (0.94%)
Chinese probands with inherited or state unknown variants (*n* = 1102)	345 (31.31%)	49 (4.45%)	Subtotal: 394 (35.75%)

*PTCHD1* and *ARHGEF9* were in two groups, including FDR_TADA_combined_ < 0.05 and genes with X-linked hemizygous

### Functional Characteristics of Prioritized Candidate Genes

We next employed MetaScape [58] to perform functional enrichment analysis. As expected, these candidate genes were significantly enriched in NDD-associated pathways, such as synapse organization, covalent chromatin modification, head development, behavior, and regulation of ion transport [17, 59–63] (Fig. S3). Interestingly, the novel candidate genes were also involved in similar biological pathways. For example, *MSL2* was implicated in covalent chromatin modification, *LRRC4* was involved in chemical synaptic transmission, and *PLXNA3* was associated with head development and axonogenesis.

We also performed functional cell-specific enrichment analyses [64, 65] to investigate whether candidate genes were associated with specific tissues or cells. By analyzing the mouse transcriptomic profiling datasets of different developmental stages and brain regions, we found that the 212 NDD candidate genes tended to be enriched in the cortex and striatum during the middle fetal stage (Fig. S4a). In the cell-specific enrichment analyses, we observed a highly significant enrichment in Drd1 + and Drd2 + spiny neurons of neostriatum and rods (Fig. S4b), similar to data reported in a previous study [21]. These results suggest that the 212 NDD candidate genes are functionally associated with the etiology of NDDs.

### Functional Network Analysis Between Known and Novel Candidate Genes

To investigate correlations between novel and known candidate genes, we performed a permutation test to estimate the relationship between these genes based on co-expression gene pairs identified from the BrainSpan atlas. We found that 15 of the 17 novel candidate genes (*p* = 2.35 × 10^−3^, permutation test, Fig. S5a) were co-expressed with 285 known candidate genes (*p* = 3.72 × 10^−4^, permutation test, Fig. S5b), with 523 connections between them (*p* = 8.80 × 10^−5^, permutation test, Fig. S5c), suggesting that the novel candidate genes are significantly co-expressed with the known candidate genes and are more likely to be related to the pathology of NDDs.

**Fig. 2:**
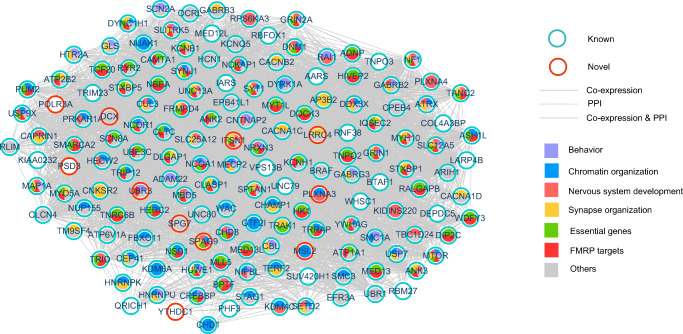
Functional network of novel and known candidate genes. Based on co-expression data from BrainSpan and PPI data from IntAct, 159 candidate genes formed a large interconnected functional network, mainly involving the following major functional clusters: chromatin organization, essential genes, nervous system development, FMRP targets, behavior, and synapse organization. Novel candidate genes are in red circles and known candidate genes are in green circles. Different line types between nodes represent the interactions existing in BrainSpan or IntAct or in both BrainSpan and IntAct

To further investigate the functional relationship between novel and known candidate genes, we constructed a functional network by integrating PPI data from IntAct and brain expression data from BrainSpan. Only known candidate genes directly interacting/co-expressed with at least two novel candidate genes were added to the network. The co-expressed/PPI network encompassed 159 genes, including 11 novel candidate genes and 148 known candidate genes (Fig. 2). These genes were enriched in several biological processes known to be related to NDDs, such as covalent chromatin modification (GO:0016569, *p* = 2.35 × 10^−12^, Fisher’s exact test), chemical synaptic transmission (GO:00072686, *p* = 7.31 × 10^−12^, Fisher’s exact test), and brain development (GO:0007420, *p* = 1.23 × 10^−7^, Fisher’s exact test). Furthermore, these genes showed a significant enrichment of previously reported gene sets: FMRP targets [66] (*p* < 2.22 × 10^−16^, Fisher’s exact test), and genes essential in mice [67] (*p* < 2.22 × 10^−16^, Fisher’s exact test). It is worth noting that six novel candidate genes (*UBR3*, *PLXNA3*, *ITSN1*, *MSL2*, *LRRC4*, and *SPAG9*) were significantly involved in functional clusters known to be associated with NDDs. For example, *MSL2*, *UBR3*, *SPAG9*, and *PLXNA3* are involved in chromatin organization, while *ITSN1*, *SPAG9*, and *PLXNA3* have been implicated in nervous system development. In addition, we found that nine novel candidate genes showed co-expression/interaction with more than two known candidate genes (Fig. S6). For example, *POLR3A*, which was the most frequently connected novel gene, was co-expressed/interacted with 99 known candidate genes. Other genes (*LRRC4*, *ITSN1*, *UBR3*, *PLXNA3*, *MSL2*, *SPAG9*, *PSD3*, and *YTHDC1*) were connected with more than five candidate genes. These results suggest that novel candidate genes are functionally associated with known risk genes and that these 11 novel candidate genes may have a stronger influence in the etiology of NDDs.

## Discussion

Previous studies have provided evidences that targeted sequencing and integrative analyses play a critical role in the discovery of novel candidate genes [21–24]. Based on targeted sequencing of 547 target genes, we identified 108 DNVs, accounting for ~ 10.91% (102/935) of our Chinese cohort. Among the genes with DNVs in our study, most genes have been reported to be associated with NDDs in previous studies, suggesting that genes identified in our Chinese cohort are relevant in other populations. Among the 108 DNVs, 60 DNVs were not recorded in public databases (including dbSNP, gnomAD, Gene4Denovo [39], and PubMed), consistent with the findings of Georgi et al., who reported that recurrently mutated amino acid sites in genes are rarely detected [68]. These results highlight the role of DNVs in the genetic heterogeneity of NDDs. In addition, we identified 21 genes with multiple DNVs present in 51 Chinese patients, and our analyses suggest that these genes display significant intolerance to damaging variants. Consistent with previous ASD study [19], *SCN2A* is the most frequent gene with multiple DNVs in the Chinese population. Compared with the known candidate genes, 20 of 21 genes with multiple DNVs were classified as known candidate genes. Notably, this study is the first to report *TCF20* (with its three de novo PTVs) as a candidate NDD gene in a Chinese cohort.

By integrating information regarding DNVs from public datasets with our results, we prioritized 212 candidate genes, confirming that combing public NDD datasets is beneficial for the discovery of candidate genes [22–24]. Consistent with our analyses of genes with multiple DNVs, the 212 identified candidate genes were more intolerant of damaging variants. In addition, we demonstrated that the 212 candidate genes are closely associated with the etiology of NDDs from the perspective of biological pathway and functional cell-specific enrichment analyses. These results suggest that most of the 212 candidate genes identified in our study truly contribute to NDDs and they are worth validating in genetic functional studies or replicating in cohorts.

In this study, we prioritized 17 novel candidate genes and revealed similar functional characteristics between these novel candidate genes and other known candidate genes. Through functional network analysis, we observed that novel candidate genes frequently interacted/were co-expressed with known candidate genes, and genes in the network were enriched in NDD-associated clusters, as described in previous studies [4, 17, 23, 69, 70]. Interestingly, six novel candidate genes were closely connected with known candidate genes and were involved in NDD-associated clusters, suggesting that these novel candidate genes are more likely to be associated with NDDs. For example, *ITSN1* (with an FDR value < 0.01 in the TADA analysis) was involved in nervous system development and synapse organization and connected with 64 known candidate genes. Jakob et al. reported that loss of the signaling scaffold intersectin 1 (*ITSN1*) in mice led to defective neuronal migration and ablates Reelin stimulation of hippocampal long-term potentiation [71]. Our analyses revealed that *SPAG9* carried a de novo PTV in our cohort and it is reportedly overexpressed in human astrocytoma which arises from neural progenitor cells in the central nervous system [72]. We also found that *SPAG9* was implicated in chromatin organization and co-expressed/interacted with eight known candidate genes, further supporting a role for this gene in NDDs. Among the novel candidate genes, *MSL2* was a particularly interesting gene that carried two de novo PTVs in our Chinese cohort. Except for the multiple DNVs in *MSL2*, it was the gene that frequently co-expressed/interacted with known candidate genes and was involved in the functional cluster of chromatin organization. Iossifov et al. reported a de novo *MSL2* PTV in a patient with autism [69], further suggesting that *MSL2* may be strongly linked to NDDs. We expect to have a large cohort study or functional experiments to validate this possibility in future studies.

Despite our best efforts to comprehensively integrate public data with our cohort data to discover novel risk genes, our study was still bound by some limitations. First, while we discovered that novel candidate genes identified in our study are functionally associated with known candidate genes at multiple levels, these novel candidate genes lack strong evidences that support their roles in NDDs. We expect that these novel candidate genes could be validated in larger cohorts or through functional genetic experiments. Second, the sample size of our Chinese cohort is not large enough, and future studies need to recruit more volunteers to improve the statistical power and allow for comparisons with variant patterns of other populations. Third, since NDDs are characterized by high clinical and genetic heterogeneity, and multiple risk factors contribute to the etiology of these disorders, candidate gene analyses can only partially elucidate the processes underlying NDDs. Further studies are needed to integrate the impact of distinct influences such as epigenetic, environmental, and genetic factors.

In summary, our study demonstrates that integrating DNVs from multiple NDD-related studies can help in the identification of risk genes. We highlight that the pattern of DNVs in Chinese cohorts is relevant to other populations. Furthermore, we provide evidence at multiple levels that novel candidate genes are functionally associated with known candidate genes. Finally, our study describes new high-confidence risk genes that should aid the study of the NDD etiology and we expect that these genes will be worth analyzing in large cohorts or being validated in genetic functional experiments.

## Supplementary Information

ESM 1 (PDF 147 kb)

ESM 2 (PDF 1817 kb)

ESM 3 (PDF 584 kb)

ESM 4 (PDF 1668 kb)

ESM 5 (PDF 358 kb)

ESM 6 (PDF 96 kb)

ESM 7 (XLSX 49 kb)

ESM 8 (XLSX 269 kb)

ESM 9 (XLSX 83 kb)

ESM 10 (XLSX 23 kb)

ESM 11 (XLSX 12 kb)

ESM 12 (XLSX 174 kb)

ESM 13 (XLSX 156 kb)

ESM 14 (XLSX 322 kb)

ESM 15 (XLSX 81 kb)

ESM 16 (DOCX 21 kb)

## Abbreviations

ASD: Autism spectrum disorder
Dmis: Deleterious missense variant
DNV: De novo variant
FDR: False discovery rate
NDD: Neurodevelopmental disorder
PPI: Protein-protein interaction
PTV: Protein-truncating variant
RIV: Rare inherited variant
WES: Whole-exome sequencing
WGS: Whole-genome sequencing
