# Supplementary figures and images for: Targeted sequencing and integrative analysis to prioritize candidate genes in neurodevelopmental disorders
Source: Mol Neurobiol. 2021 Apr 15;58(8):3863–73. doi: 10.1007/s12035-021-02377-y (PMC8280036; doi:10.1007/s12035-021-02377-y)

a

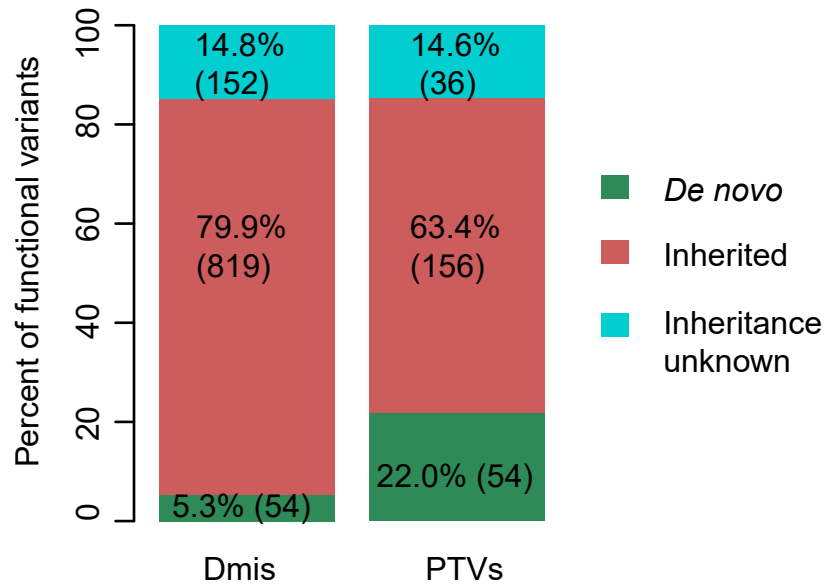

b

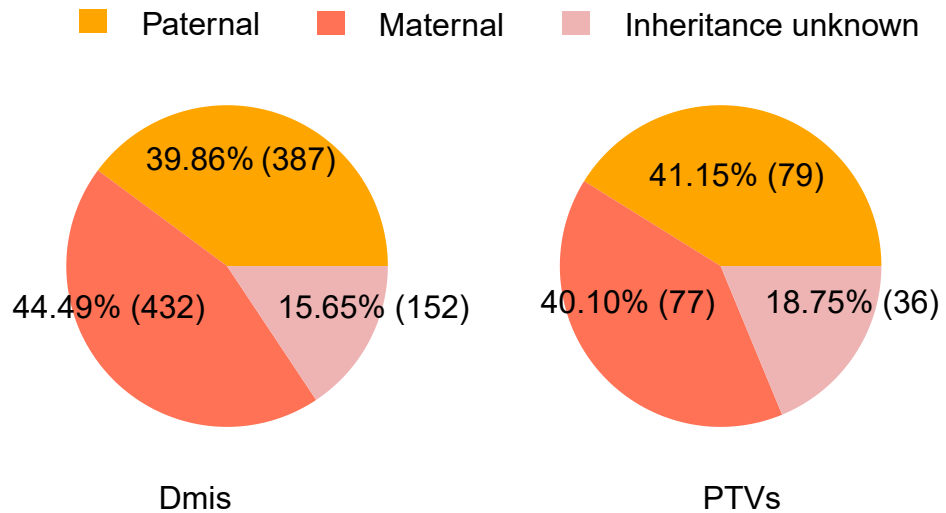

Supplement: Supplementary file 1 — (PDF 147 kb) [file 12035_2021_2377_MOESM1_ESM.pdf]

a

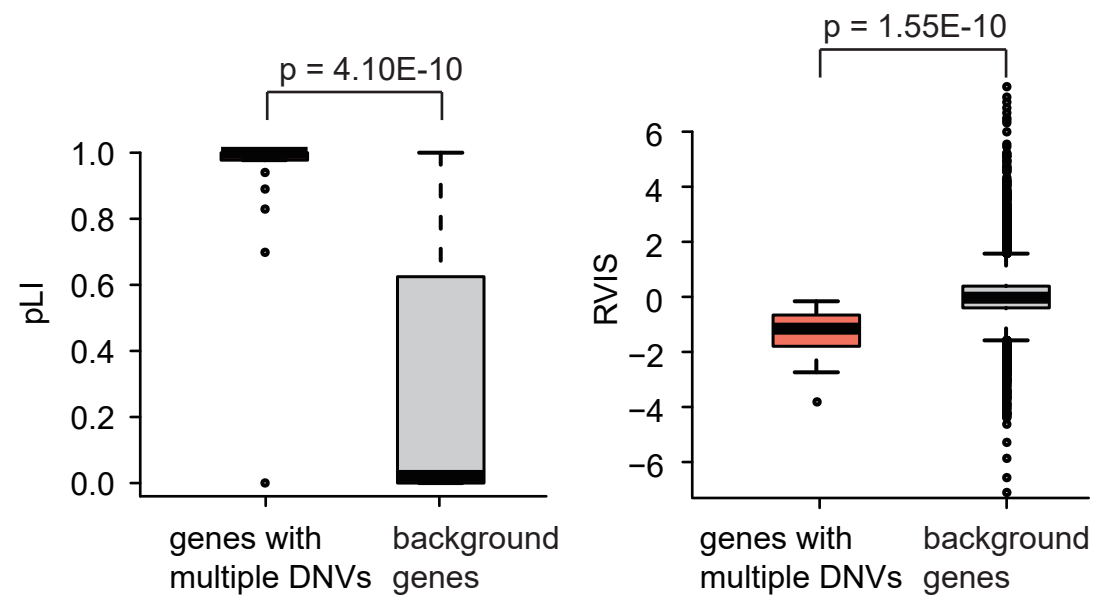

b

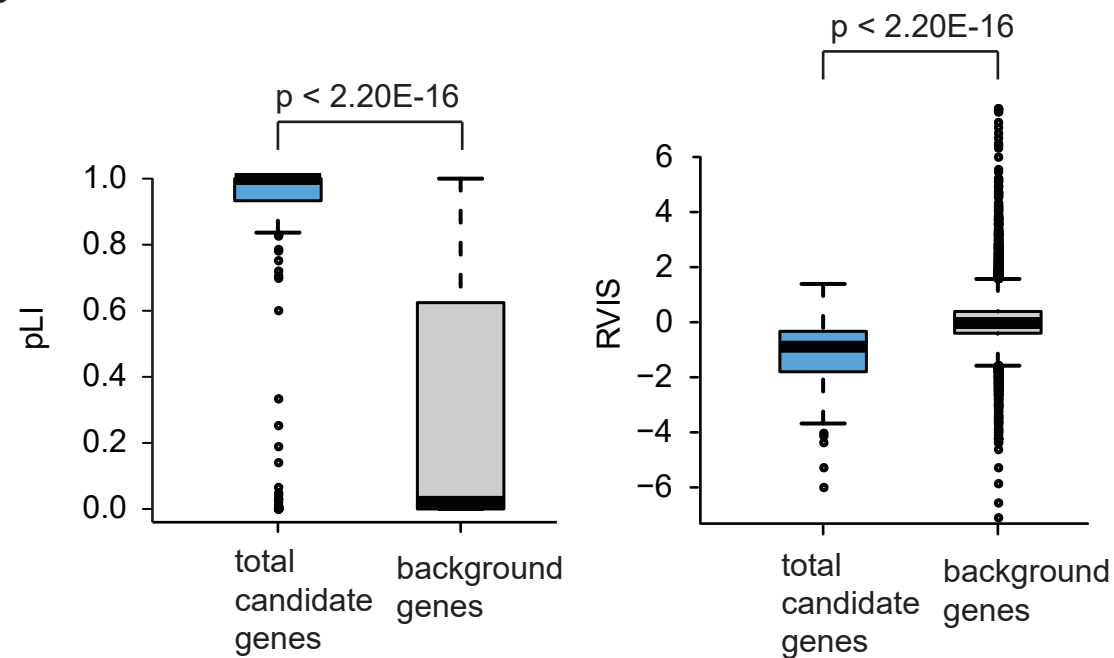

c

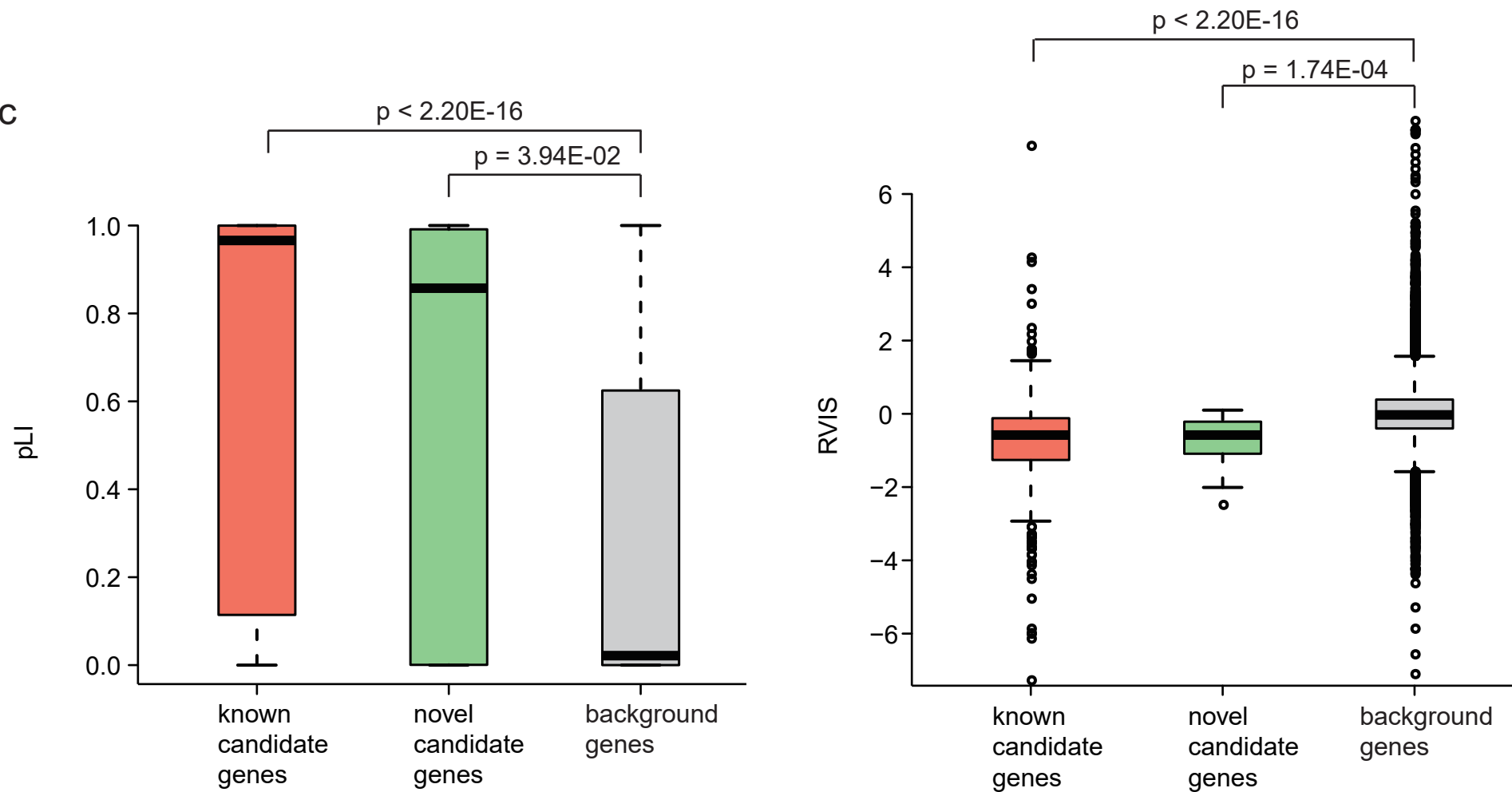

Supplement: Supplementary file 2 — (PDF 1817 kb) [file 12035_2021_2377_MOESM2_ESM.pdf]

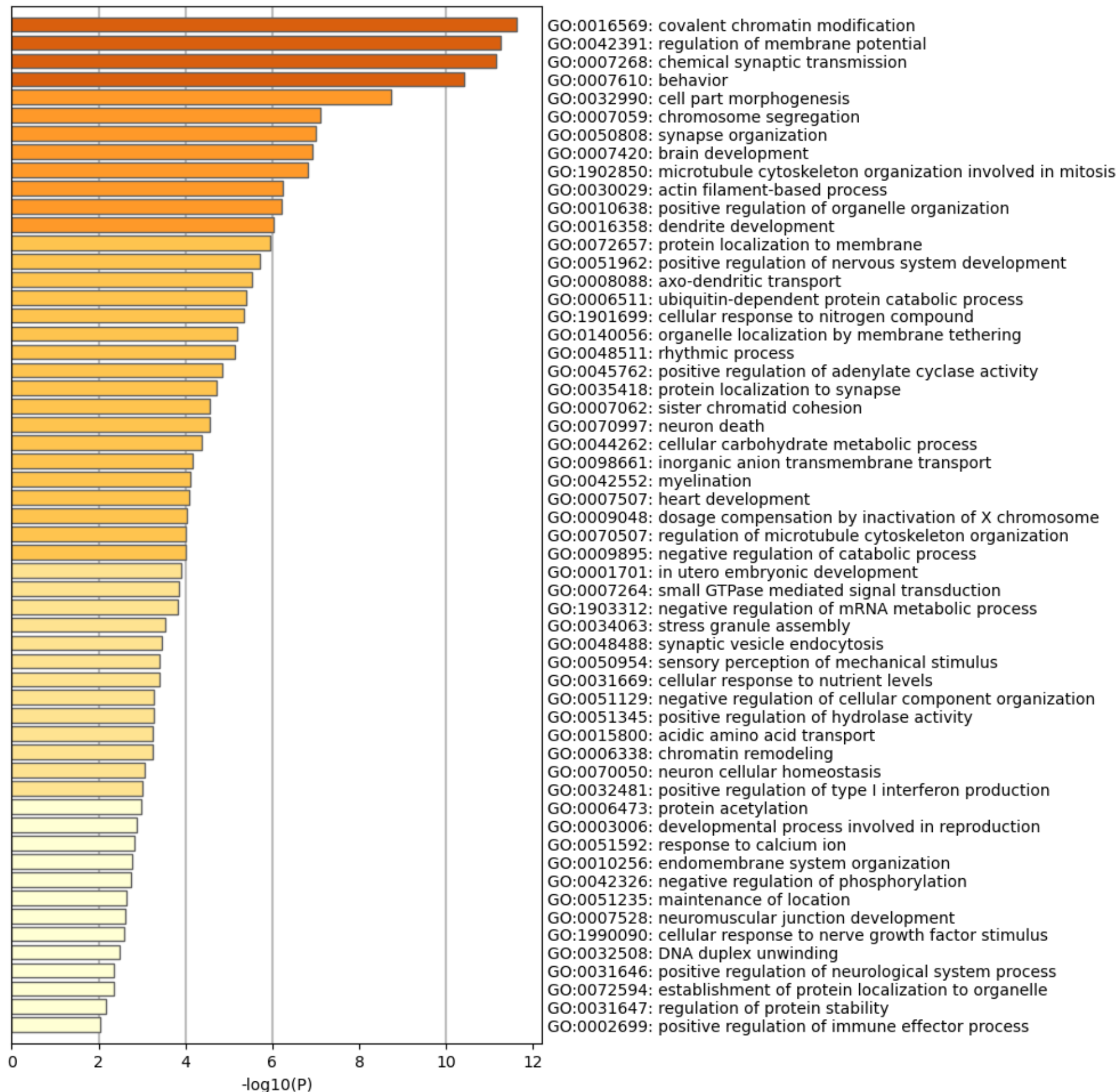

Supplement: Supplementary file 3 — (PDF 584 kb) [file 12035_2021_2377_MOESM3_ESM.pdf]

a

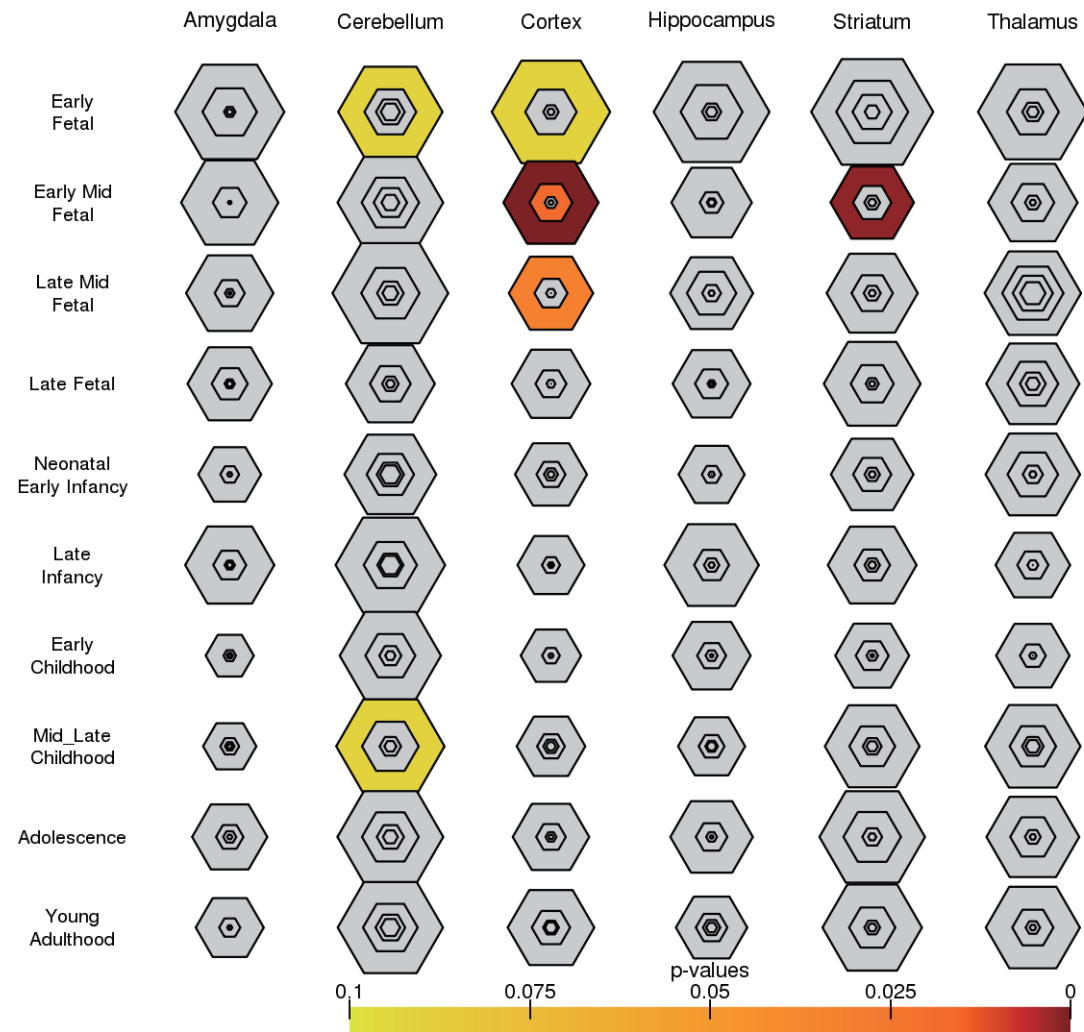

**b**

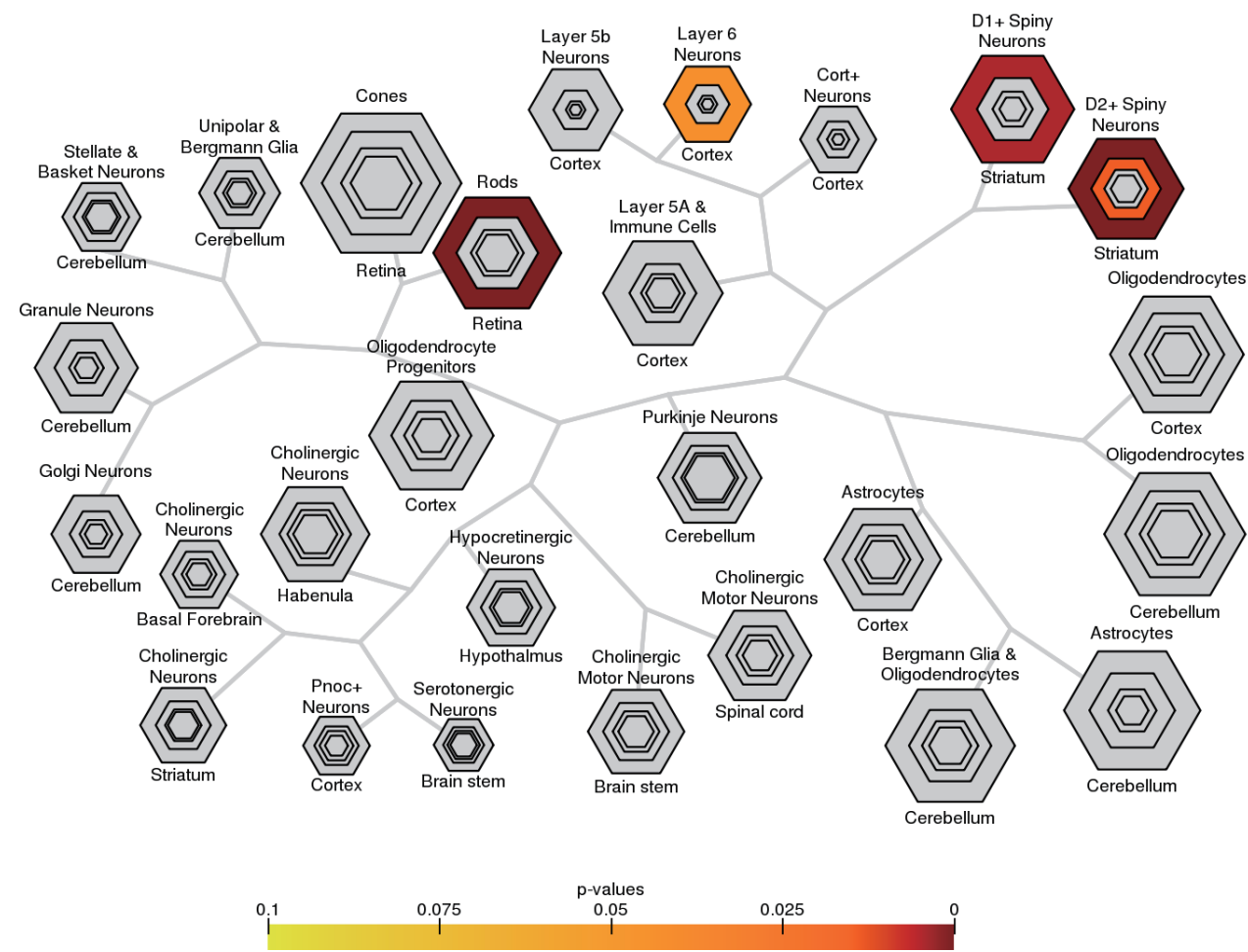

Supplement: Supplementary file 4 — (PDF 1668 kb) [file 12035_2021_2377_MOESM4_ESM.pdf]

a

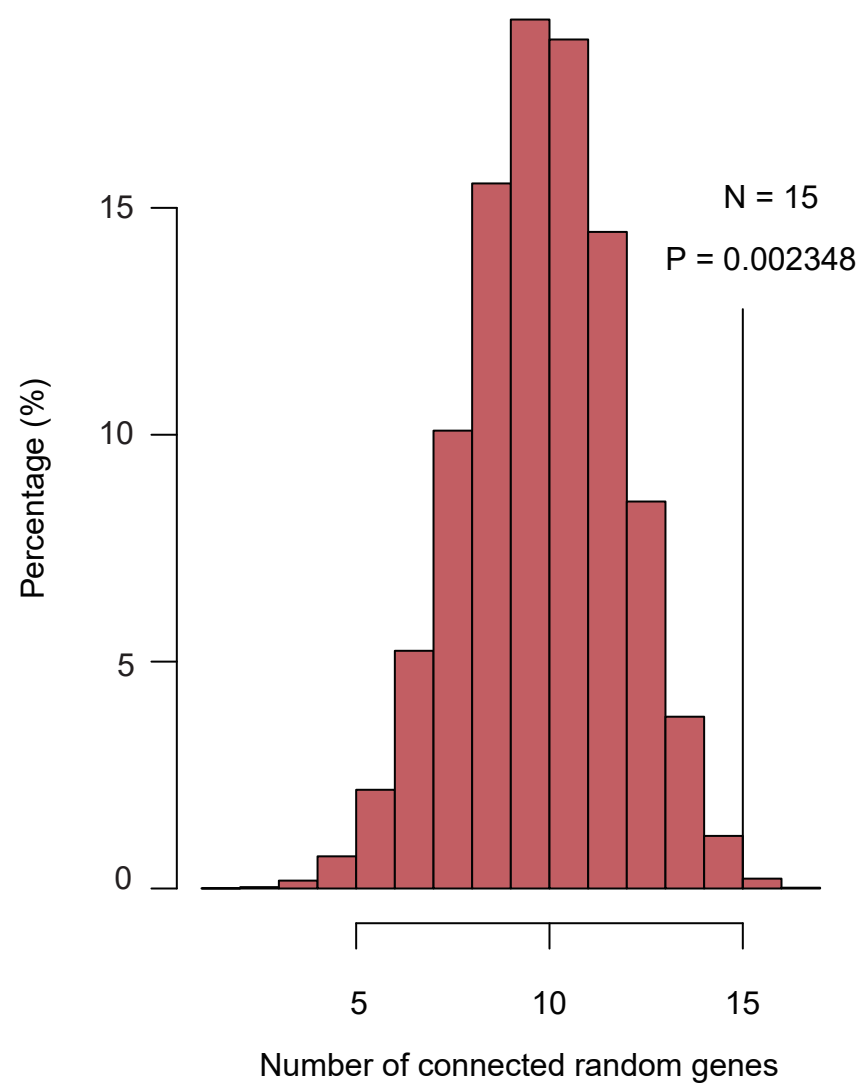

b

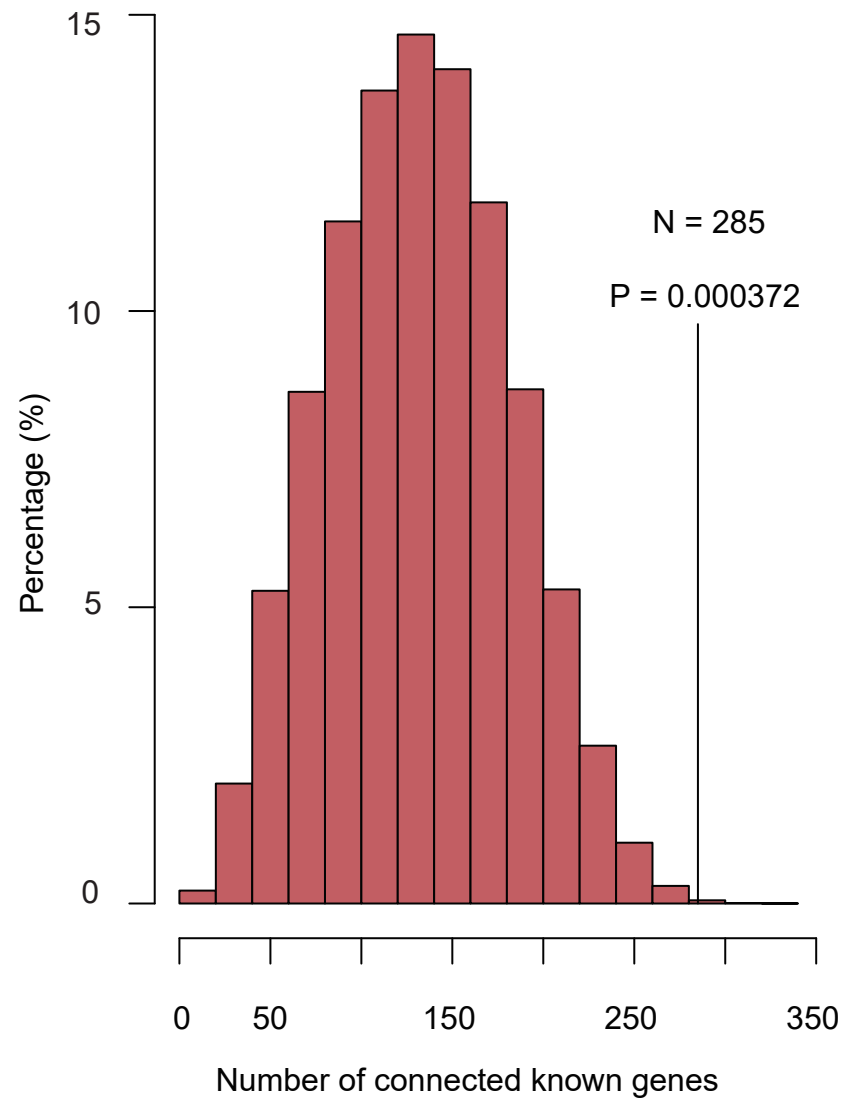

c

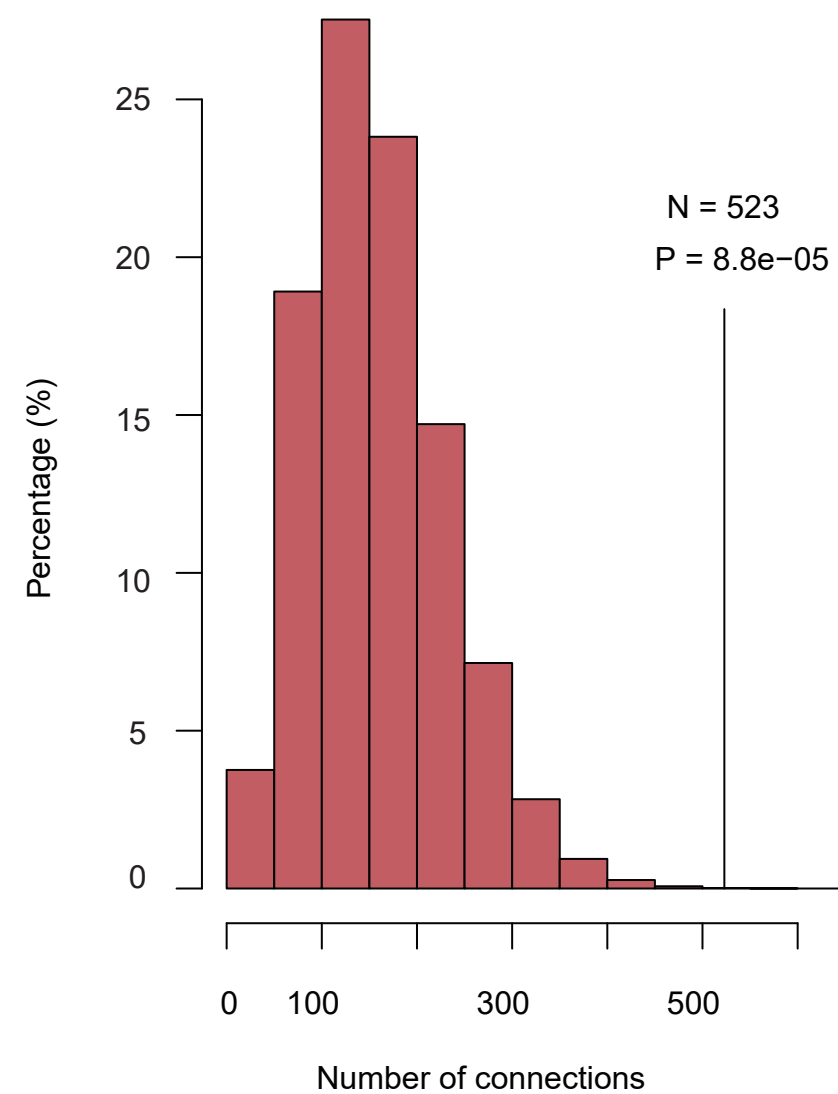

Supplement: Supplementary file 5 — (PDF 358 kb) [file 12035_2021_2377_MOESM5_ESM.pdf]

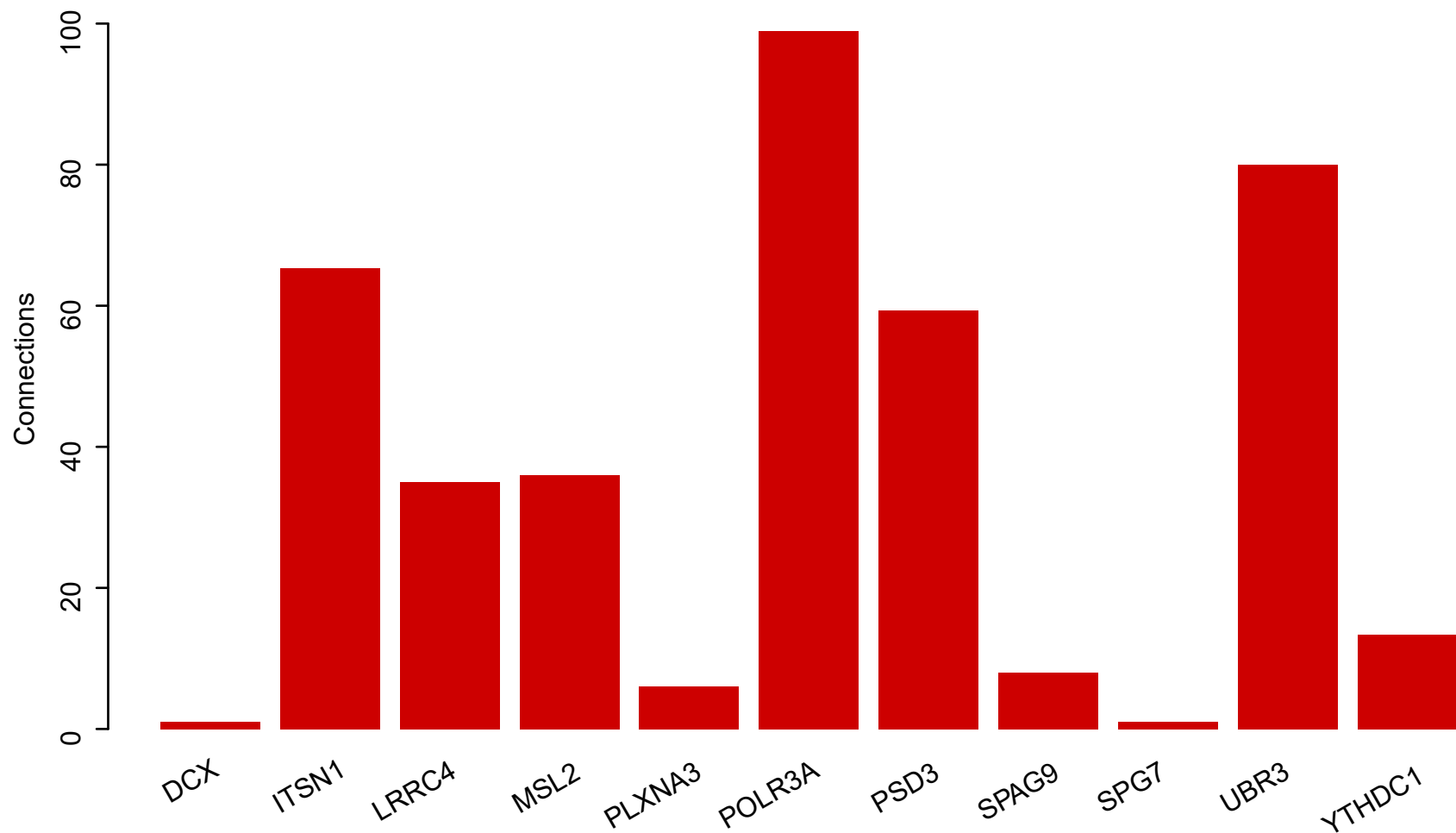

Supplement: Supplementary file 6 — (PDF 96 kb) [file 12035_2021_2377_MOESM6_ESM.pdf]
