# Supplementary material for: Targeted sequencing and integrative analysis to prioritize candidate genes in neurodevelopmental disorders
Source: Mol Neurobiol. 2021 Apr 15;58(8):3863–73. doi: 10.1007/s12035-021-02377-y (PMC8280036; doi:10.1007/s12035-021-02377-y)
Supplement: Supplementary file 16 — (DOCX 21 kb) [file 12035_2021_2377_MOESM16_ESM.docx]

Supplementary Materials

**Figure S1. The summary of variants identified in our study.**

a. The distribution of deleterious missense variants and protein-truncating variants. b. The distribution of inherited variants.

**Figure S2. pLI and RVIS distributions for three subtypes of candidate genes.**

a. pLI and RVIS distributions for genes with multiple DNVs. b. pLI and RVIS distributions for total candidate genes. c. pLI and RVIS distributions for known candidate genes and novel candidate genes. P value was calculated by Wilcoxon rank sum test.

**Figure S3. Enriched pathways for 212 NDD-candidate genes.**

The enrichment analysis of 212 NDD-candidate genes. The darker the color, the smaller the *p* value. Each bar represents one enriched pathway. P values were calculated by Fisher’s exact test.

**Figure S4. Cell-specific enrichment analysis of 212 NDD-candidate genes.**

a. Specific expression analysis across brain regions and development stages. b. Specific expression analysis across cell types.

**Figure S5. Permutation test of connections between novel and known candidate genes.**

The solid line represents expected connected genes. P values were calculated by permutation test.

**Figure S6. Connections of novel candidate genes in the functional network.**

The horizontal axis shows the novel candidate genes in functional network. The vertical axis shows the connections of novel candidate genes.

**Table S1. 574 target genes.**

The detailed information of 574 target genes in SFARI and AutismKB database. SFARI_gene-score: gene scores in SFARI database, 1 represents high confidence, 2 represents strong candidate, 3 represents suggestive evidence, - represents no records. SFARI_syndromic: 1 represents that genes are in the syndromic category, 0 represents that genes are not in the syndromic category. SFARI_number-of-reports: total reports in SFARI database. SFARI_number-autism-reports: total autism-associated reports in SFARI database. AutismKB_Core_dataset: Y represents genes in AutismKB core dataset, N represents genes not in AutismKB core dataset but recorded in AutismKB, - represents genes not recorded in AutismKB. AutismKB_syndromic_disorder: associated syndromic disorders. AutismKB_total_score: total score in AutismKB.

**Table S2. Full list of 1,271 sanger validated variants in our Chinese cohort.**

ReVe: the predictive score of missense variants. Deleterious missense variants with ReVe score > 0.7. D represents deleterious missense variants, T represents tolerant variants. gnomAD_exome_ALL: allele frequency of all populations in exome sequencing data. gnomAD_exome_EAS: allele frequency of East Asian in exome sequencing data. gnomAD_genome_ALL: allele frequency of all populations in genome sequencing data. gnomAD_genome_EAS: allele frequency of East Asian in genome sequencing data.

**Table S3. Collected known candidate genes of NDDs.**

SFARI_gene-score: gene scores in SFARI database, 1 represents high confidence, 2 represents strong candidate, 3 represents suggestive evidence, - represents no records. SFARI_syndromic: 1 represents that genes are in the syndromic category, 0 represents that genes are not in the syndromic category. SFARI_number-of-reports: total reports in SFARI database. SFARI_number-autism-reports: total autism-associated reports in SFARI database. OMIM_Phenotypes: phenotypes recorded in OMIM database.

**Table S4. Detailed information of 21 genes with multiple *de novo* PTVs in our Chinese cohort.**

gnomAD_exome_ALL: allele frequency of all populations in exome sequencing data. gnomAD_genome_ALL: allele frequency of all populations in genome sequencing data. SFARI_gene-score: gene scores in SFARI database, 1 represents high confidence, 2 represents strong candidate, 3 represents suggestive evidence, - represents no records. SFARI_syndromic: 1 represents that genes are in the syndromic category, 0 represents that genes are not in the syndromic category. SFARI_number-of-reports: total reports in SFARI database. SFARI_number-autism-reports: total autism-associated reports in SFARI database. OMIM_Phenotypes: phenotypes recorded in OMIM database.

**Table S5. Detailed information of six genes with hemizygous variants in our probands.**

gnomAD_exome_ALL: allele frequency of all populations in exome sequencing data. gnomAD_genome_ALL: allele frequency of all populations in genome sequencing data. TADA_FDR_Our_data: FDR calculated by our data. TADA_FDR_Our_and_Public_NDD_data: FDR calculated by our data and public data. Novel gene screening (PMID): Known represents known candidate genes, Novel represents novel candidate genes. SFARI_gene-score: gene scores in SFARI database, 1 represents high confidence, 2 represents strong candidate, 3 represents suggestive evidence, - represents no records. SFARI_syndromic: 1 represents that genes are in the syndromic category, 0 represents that genes are not in the syndromic category. SFARI_number-of-reports: total reports in SFARI database. SFARI_number-autism-reports: total autism-associated reports in SFARI database. OMIM_Phenotypes: phenotypes recorded in OMIM database.

**Table S6. Integrated PTVs in 3582 Chinese controls.**

Sample_Hom**:** total homozygous samples in 3582 contrsls; Sample_Het: total heterozygous samples in 3582 controls; gnomAD_exome_ALL: allele frequency of all populations in exome sequencing data. gnomAD_genome_ALL: allele frequency of all populations in genome sequencing data.

**Table S7. Detailed result of TADA analysis.**

The detailed TADA results in our Chinese cohort, published data, and a combination of both. Both p value and FDR were calculated in TADA model. Mut.rate.PTV: the mutation rate of PTV. Mut.rate.Dmis: the mutation rate of deleterious missense mutations. dn.PTV: the number of *de novo* PTV in 935 trios. dn.Dmis: the number of *de novo* Dmis in 935 trios. case.PTV: the number of inherited PTV in our Chinese probands. control.PTV: the number of inherited PTV in inhouse Chinese controls.

**Table S8. Full list of 2,147 functional *de novo* variants in 16,807 NDD probands collected from Gene4Denovo.**

gnomAD_exome_ALL: allele frequency of all populations in exome sequencing data. gnomAD_genome_ALL: allele frequency of all populations in genome sequencing data. Patient_ID: patient id recorded in Gene4Denovo.

**Table S9. Full list of 208 candidate genes prioritized by TADA analysis at FDR less than 0.1.**

Both p value and FDR were calculated in TADA model. Mut.rate.PTV: the mutation rate of PTV. Mut.rate.Dmis: the mutation rate of deleterious missense mutations. dn.PTV: the number of *de novo* PTV in 935 trios. dn.Dmis: the number of *de novo* Dmis in 935 trios. case.PTV: the number of inherited PTV in our Chinese probands. control.PTV: the number of inherited PTV in inhouse Chinese controls.
